# Self-Guided Algorithm for Fast Image Reconstruction in Photo-Magnetic Imaging: Artificial Intelligence-Assisted Approach

**DOI:** 10.3390/bioengineering11020126

**Published:** 2024-01-28

**Authors:** Maha Algarawi, Janaki S. Saraswatula, Rajas R. Pathare, Yang Zhang, Gyanesh A. Shah, Aydin Eresen, Gultekin Gulsen, Farouk Nouizi

**Affiliations:** 1 Department of Physics, College of Science, Imam Mohammad Ibn Saud Islamic University (IMSIU), Riyadh 13318, Saudi Arabia; mmgarawi@imamu.edu.sa; 2 Tu and Yuen Center for Functional Onco-Imaging, Department of Radiological Sciences, University of California Irvine, Irvine, CA 92697, USA; jsaraswa@uci.edu (J.S.S.); rrpathar@uci.edu (R.R.P.); yangz17@uci.edu (Y.Z.); gyaneshs@uci.edu (G.A.S.); aeresen@hs.uci.edu (A.E.); ggulsen@uci.edu (G.G.); 3 Chao Family Comprehensive Cancer Center, University of California Irvine, Irvine, CA 92697, USA

**Keywords:** photo-magnetic imaging, inverse problems, artificial intelligence, neural network, linear regression

## Abstract

Previously, we introduced photomagnetic imaging (PMI) that synergistically utilizes laser light to slightly elevate the tissue temperature and magnetic resonance thermometry (MRT) to measure the induced temperature. The MRT temperature maps are then converted into absorption maps using a dedicated PMI image reconstruction algorithm. In the MRT maps, the presence of abnormalities such as tumors would create a notable high contrast due to their higher hemoglobin levels. In this study, we present a new artificial intelligence-based image reconstruction algorithm that improves the accuracy and spatial resolution of the recovered absorption maps while reducing the recovery time. Technically, a supervised machine learning approach was used to detect and delineate the boundary of tumors directly from the MRT maps based on their temperature contrast to the background. This information was further utilized as a soft functional a priori in the standard PMI algorithm to enhance the absorption recovery. Our new method was evaluated on a tissue-like phantom with two inclusions representing tumors. The reconstructed absorption map showed that the well-trained neural network not only increased the PMI spatial resolution but also improved the accuracy of the recovered absorption to as low as a 2% percentage error, reduced the artifacts by 15%, and accelerated the image reconstruction process approximately 9-fold.

## 1. Introduction

Diffuse optical imaging (DOI) is mainly used to quantify absolute tissue chromophore concentrations [1,2]. This allows for the estimation of key physiological parameters such as total hemoglobin and oxygen saturation, which can be used to distinguish diseased and normal tissue [3]. DOI uses light in the near-infrared (NIR) range, which covers wavelengths between 600 and 1000 nm. In this spectral window, biological tissues exhibit relatively weak absorption, allowing imaging through several centimeters of a sample [4]. Diffuse optical tomography (DOT) is a DOI technique enabling spatially-resolved functional imaging of tissue [5,6]. Although it has shown great potential in several applications such as breast cancer monitoring [7,8,9] and functional brain imaging [10,11,12,13], its translation into clinical practice has encountered impediments, primarily attributed to its inherent poor spatial resolution limitation.

Several factors have been identified as a direct cause of DOT’s poor spatial resolution and quantitative accuracy. Among these factors are the inherently ill-posed and under-determined nature of its inverse problem and the non-uniqueness of its solution, resulting from the constraint of data acquisition solely at the boundary of the imaged medium [14]. Extensive efforts have been made to overcome these limitations by combining DOT with anatomical imaging modalities such as magnetic resonance imaging (MRI), ultrasound, or X-ray computed tomography (CT) to leverage their high spatial resolution in order to recover higher quality DOT images [15,16,17]. This hybrid approach showed significant improvement but remains limited when the functional and anatomical information does not correlate.

We previously introduced photo-magnetic imaging (PMI), which is an alternative methodology that synergically combines DOI and MRI [18]. PMI provides the high resolution spatially resolved optical absorption coefficient of the tissue under investigation. The PMI data acquisition consists of the monitoring of the internal spatiotemporal distribution of the temperature variation resulting from the local absorption of photons when the tissue is irradiated with a NIR laser light. PMI is able to achieve higher spatial resolution than conventional DOI methods thanks to the utilization of magnetic resonance thermometry (MRT) for the measurement of the laser-induced internal spatiotemporal temperature [19,20]. Following data acquisition, the high-resolution optical absorption maps are obtained using a dedicated PMI reconstruction algorithm [21,22]. The image reconstruction is based on the minimization of the difference between the measured and synthetic spatiotemporal temperature maps, which are simulated by solving the combined diffusion and Pennes’ bio-heat equations using the finite element method (FEM) [23,24,25]. This conventional FEM-based algorithm performs highly but remains limited by its relatively long computation time.

The acceleration of the PMI image reconstruction algorithm is possible either through the acceleration of the resolution of the forward or the inverse problems. We previously decreased the resolution time of the forward problem by implementing an analytical approach that generates fast and accurate solutions to the combined diffusion and Pennes’ bio-heat equations [26,27,28,29,30]. On the other hand, the resolution of the inverse problem is performed using conventional optimization methods such the gradient descent or algebraic reconstruction technique (ART). Accelerating its resolution would necessitate the use of additional information that can improve the convergence quality of these optimization methods. During the PMI MRT image acquisition, we could observe that cancerous lesions warmed up slightly higher compared to the surrounding normal tissue. This was due to their higher absorption of the NIR photons, resulting from their higher hemoglobin concentration. Thus, extracting geometrical characteristics about these cancerous lesions directly from the MRT maps and using them to guide and constrain the optimization step would theoretically accelerate the resolution of the inverse problem. Unfortunately, the MRT image blurring caused by the heat diffusion makes delineating the accurate boundaries of these lesions directly on the MRT maps complicated, and is not a straight forward process [18].

In this paper, we present an artificial intelligence (AI)-driven algorithm that overcomes the heat diffusion blurring effect. It allows for the precise recovery of accurate boundaries of the cancerous lesions within the tissue directly from the MRT maps and prior to any reconstruction process. These boundaries are used to implement a binary mask that will be used to improve the preconditioning of the PMI image reconstruction algorithm, and thus accelerate the convergence of its minimization problem. Our AI-driven approach operates as a two-stage detection network, integrating machine learning (ML) and multi-linear regression techniques. In the initial stage, a convolutional neural network is deployed to identify regions within the MRT maps exhibiting higher temperature increases indicative of the presence of cancerous lesions. The identified regions are then fed to the second stage of the algorithm to generate a binary mask, effectively demarcating pixels that are on the cancerous regions from the ones that did warm up only due to heat diffusion. This process is performed using a statistical regression model. This innovative two-stage process not only improves the efficiency of the PMI reconstruction algorithm, but also accelerates the convergence of its minimization problem, reducing the overall reconstruction time.

## 2. Materials and Methods

### 2.1. PMI Methodology

**Figure 1:**
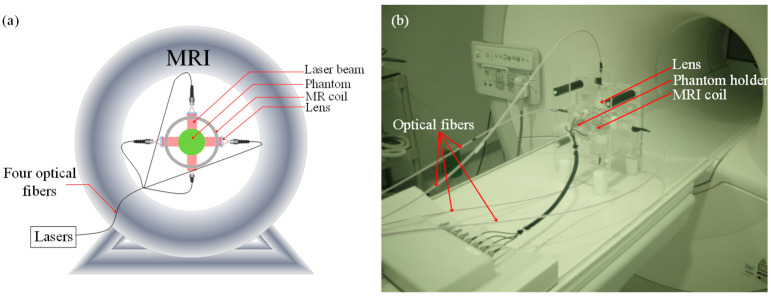
(**a**) Schematic of the PMI setup showing the phantom and the optical instrumentation inside the MRI bore. (**b**) Picture of the PMI interface sitting on the MRI bed. It consists of a specially designed RF coil with four windows for illumination and four ports that hold the collimation optics.

A Philips 3 Tesla Achieva system was used to acquire the MRT temperature maps. The phase maps were acquired using a gradient echo sequence using a 60 ms repetition time (TR) and 12 ms echo time (TE). The phantom was placed inside a home-built MRI coil paced within the MR bore (Figure 1). This imaging interface consisted of a small animal dedicated RF coil with four windows, which permitted illuminating the phantom from four sides. The phantom was illuminated using four laser diodes (780 nm, 7 W, Focuslight, Xi’an, China). Four 15-m long optical fibers were used to transport the laser light from the laser system located in the control room to the PMI interface located inside the MR bore. For in vivo imaging, the laser power per unit area was set to the ANSI limits (0.32 W/cm^2^).

Once the phantom or the small animal is positioned inside the PMI imaging interface, the internal temperature maps are acquired using a gradient echo imaging sequence as a series of phase maps [31]. Each of these phase maps is acquired in six seconds. First, while the laser is still off, a first phase map is acquired and used as the baseline (ϕ_0_). Then, the lasers are turned on and a second frame (ϕ_1_) is acquired. The increase in temperature is then a simply calculated function of the difference between the baseline map (ϕ_0_) and the second map (ϕ_1_).

### 2.2. PMI Image Reconstruction Algorithm

To reconstruct high-resolution optical absorption maps from the measured MRT ones, the PMI image reconstruction algorithm uses a gradient descent scheme to minimize the difference between these maps and the simulated temperature distributions within the medium. The simulated temperature maps are generated by modeling the propagation of laser light, and the consequent temperature increase throughout the medium using a finite element method (FEM)-based solver. This step is commonly known as the resolution of the forward problem.

#### 2.2.1. PMI Forward Problem

The PMI forward problem resolution is performed in two steps. Firstly, the propagation of light in the medium (Ω) is modeled using the diffusion equation [32]. Technically, this step calculates the density of photons Φ(r)[W mm^−2^] at any position r [mm] using the spatial distribution $\mu_{a}$[mm^−1^] and D [mm] of the absorption and diffusion coefficients, respectively. The diffusion coefficient D is defined as $D\left(r\right)=1/3(\mu_{a}$+$\mu_{s}^{\prime }$) with $\mu_{s}^{\prime }$ [mm^−1^] being the reduced scattering coefficient.

(1) $$\left\{\begin{array}{c} -\nabla \cdot \left[D\left(r\right)\nabla \Phi \left(r\right)\right]+\mu_{a}\left(r\right)\Phi \left(r\right)=q_{0}\left(r\right),(r\in \Omega )\\ \vec{n}\cdot D\nabla \Phi (r)+A\Phi (r)=0(r\in \delta \Omega ) \end{array}\right.$$

where $q_{0}(r)$ is the isotropic source of light, ∇ denotes the gradient operator, δΩ is the surface boundary, $\vec{n}$ is the vector normal to δΩ, and *A* is the coefficient modeling the surface mismatch [33].

Secondly, the laser-induced increase in temperature T[°C] and its dynamics T*(r,t)* within the medium are modeled using the Pennes bio-heat equation [34]:

(2) $$\left\{\begin{array}{l} \rho c\frac{\partial T(r,t)}{\partial t}-\nabla \cdot \left[k\nabla T(r,t)\right]=\Phi (r)\mu_{a}(r),(r\in \Omega )\\ -k\frac{\partial T(r,t)}{\partial r}=h\left[T_{f}(r)-T(r)\right](r\in \delta \Omega ) \end{array}\right.$$

where ρ [g mm^−3^] is the density, c [J (g °C)^−1^] is the specific heat, and *k* [W (mm °C)^−1^] is the thermal conductivity of the medium. The source of heat resulting from the laser light absorption by the medium is modeled by the product of the optical absorption and the photon density at any point within the medium [35,36]. $T_{f}$[°C] is the ambient temperature and h [W (mm^2^ °C)^−1^] is the heat transfer coefficient at the surface of the medium.

#### 2.2.2. PMI Inverse Problem

The resolution of the PMI inverse problem is achieved by iteratively minimizing the quadratic difference between the measured, $T^{m}$, and the simulated, $T(\mu_{a})$, temperatures as follows:

(3) $$O\left(\mu_{a}\right)=\sum_{d=1}^{N_{D}}\;{∥T_{s,d}^{m}-T_{s,d}\left(\mu_{a}\right)∥}^{2}$$

where $N_{D}$ denotes the number of detectors. Since MRT allows one to measure the temperature at any position within the medium, $N_{D}$ is equal to the number of FEM mesh nodes N. During the resolution of the PMI inverse problem, the objective function is minimized while iteratively updating the unknown $\mu_{a}$ using the Levenberg–Marquardt method by [37]:

(4) $$\Delta \mu_{a}={\left(J^{T}J+\alpha I\right)}^{-1}J^{T}\left[T^{m}-T\left(\mu_{a}\right)\right]$$

where J is the Jacobian matrix, α is a regularization parameter, and I is the identity matrix.

It has been shown that use of a priori information drastically improves the image reconstruction process. Indeed, this scheme helps guide and constrain the inverse problem described in Equation (4). The a priori information, generally consisting of the boundaries of regions of interest (ROIs), is incorporated into the resolution of the inverse problem as soft a priori as follows:

(5) $$\Delta \mu_{a}={\left(J^{T}J+\alpha L^{T}L\right)}^{-1}J^{T}\left[T^{m}-T\left(\mu_{a}\right)\right]$$

where L is a penalty matrix that is implemented based on the boundaries of the identified ROIs of the imaged medium [38,39]:

(6) $$L_{ij}=\left\{\begin{array}{cc} 0 & i\;\mathrm{and}\;j\;\mathrm{not}\;\mathrm{in}\;\mathrm{the}\;\mathrm{same}\;\mathrm{region}\;\\ -\frac{1}{N_{r}} & \;i\;\mathrm{and}\;j\;\mathrm{in}\;\mathrm{the}\;\mathrm{same}\;\mathrm{region}\;\\ 1 & i=j \end{array}\right.$$

where $N_{r}$ represents the number of FEM nodes belonging to each of the ROIs.

### 2.3. AI-Based A Priori Information Generation

Generally, the a priori information is retrieved from an anatomical imaging modality that is used in tandem with the functional one [16]. Here, we introduce a new AI-based algorithm that allows for the recovery of the binary mask directly from the MRT maps. The ROIs are detected and delineated based on their higher increase in temperature compared to the background. Since the binary mask is obtained from the MRT maps and not from the MRI anatomical images, we will call it functional information henceforth. Our algorithm is based on a supervised machine learning approach. The detection of hot nodes is obtained following a two-step procedure: (1) Delineation of the region of interest and (2) Prediction of hot nodes.

#### 2.3.1. ML-Based Delineation of Region of Interest

The synthetic data needed to train our ML-based algorithm were generated on a numerical cylindrical phantom with the same geometry as the agarose phantom used in the experimental study. Its optical properties were set to mimic mouse muscle ($\mu_{a}$ = 0.01 mm^−1^, $\mu_{s}^{\prime }$ = 0.8 mm^−1^) [18]. The thermal properties of the agarose phantom are considered equivalent to water, given that the phantom comprises approximately 98% water [30]. This 25-mm diameter cylinder was used as the background geometry. Since the training data require a variety of cases, different inclusions were embedded into this background to mimic the presence of tumors. A set of 3927 cases was obtained by varying the size, location, and absorption coefficient of these inclusions [18,23,40]. Due to their higher concentration of hemoglobin, tumors are generally characterized with a higher optical absorption coefficient. Thus, the mesh nodes within inclusions were defined and their absorption was set to higher values than the background [23].

**Figure 2:**
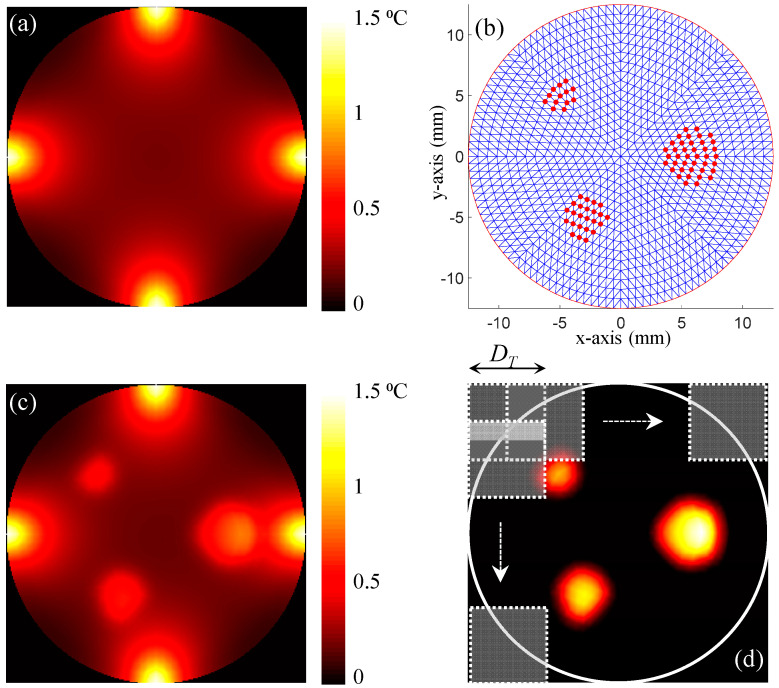
(**a**) Temperature map obtained on the homogenous phantom. (**b**) A representative 852-node FEM mesh of the 25-mm diameter circular phantom. The hot nodes are highlighted with red dots. (**c**) Temperature map obtained on the heterogenous phantom hosting three inclusions with diameters of 2.5 mm, 2 mm, and 1.5 mm, which were embedded at positions (6,0), (−3,−5), and (−5,5), respectively. (**d**) The sub-image extraction using the Tiles method on the temperature difference map. D_T_ denotes the width of the Tile. Tiles were slid by ½ D_T_ to ensure overlapping by half the tile size.

When solving the forward problem on the homogeneous phantom without the presence of any inclusion, a significant temperature rise was observed straight below the four illumination spots [26,27,30]. Then, the temperature exhibited an exponential decay with depth (Figure 2a). In order to generate a representative heterogenous phantom, three inclusions with diameters of 2.5 mm, 2 mm, and 1.5 mm were embedded at position (6,0), (−3,−5), and (−5,5), respectively. Figure 2b shows the FEM mesh and the nodes belonging to the three inclusions in red asterisks. These nodes will be referred to as hot nodes in the rest of the paper.

After heating this phantom from its four sides for eight seconds, the temperature increase exhibited a similar scheme to the one observed on the homogenous phantom (Figure 2c). However, a higher rise in temperature was observed deep inside the medium at the location of the inserted inclusions this time. This higher increase in temperature resulted from the higher optical absorption of the embedded inclusions. Nevertheless, the increase in temperature remained limited compared to the one observed below the laser spots. In order to eliminate the increase in temperature below the laser spots and emphasize the increase in temperature at the inclusions, the temperature map obtained using the homogenous was subtracted. The difference in temperature rise between the phantoms with and without the presence of the inclusions is presented in Figure 2d. This step corresponds to the difference $T^{m}-T(\mu_{a})$ in Equation (5) at the first iteration of the minimization process. These processed data were used to train our neural network.

First, the temperature maps were divided into overlapping sub-images, called “tiles”. Each tile overlapped the adjacent one with exactly half its size ($D_{T}$) in each of the directions, as shown in Figure 2d.

**Figure 3:**
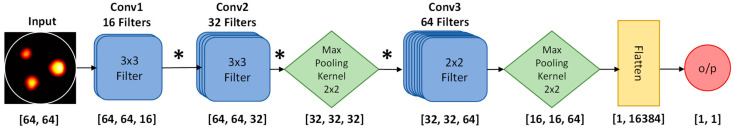
Structure of the ML classifier model used for the binary classification of the tiles. The * sign denotes the convolution operator.

Then, by mapping the tiles onto the mesh nodes, the tiles are labeled as “positive” if they contain at least one of the hot nodes, and “negative” otherwise. Henceforth, a tile and its corresponding binary label pair is referred to as the training pair. These training pairs were used to train the ML classifier model composed of six layers, as shown in Figure 3. Use of a sigmoidal nonlinearity activation function associated allows the classifier to yield a binary output. The training of our neural network was performed on 75% of the dataset using the Adam Optimizer optimization method until the accuracy exceeded 90%.

Finally, for each of the tiles classified as positive, the FEM mesh nodes contained within that tile are saved and provided as input to the second step of our method to predict which of them will be detected as hot nodes.

#### 2.3.2. Prediction of Hot Nodes

**Figure 4:**
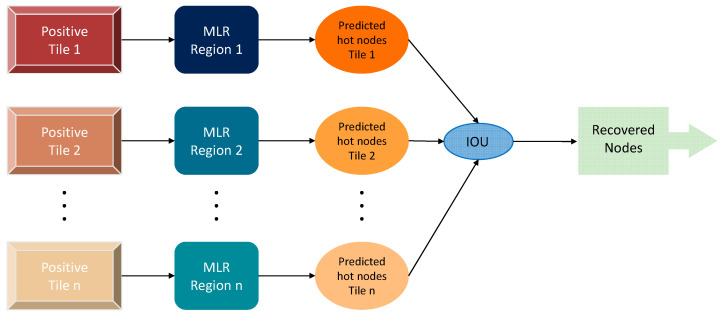
Framework of the prediction of the hot nodes. MLR, multi-linear regression model. IOU, intersection over union.

At the output of the ML classifier, only the nodes (*M*) within the tiles classified as “positive” are used for the final prediction of the hot nodes rather than the entire (*N*) nodes of the FEM mesh. This considerably reduces the computation time of the prediction of the hot nodes. Since the inclusions only represent a small part of the surface of the tile, the nodes within the area outside the inclusion are considered “cold nodes”. These nodes will be considered false positive predicted nodes if classified as hot nodes. Here, to accurately determine the hot nodes from the cold ones in each tile, we implemented a weighted scheme that allowed us to decrease these false positives and better predict the hot nodes, as can be seen in Figure 4.

For each of the phantoms, the ML-classifier was used to determine the positive tiles. Each of these positive tiles was then mapped to the FEM mesh in order to determine the *M* nodes that belonged to it. Knowing the position of the inclusion within the FEM mesh, a binary vector of size [*M* × 1] was implemented by assigning “1” to the hot nodes and “0” to the cold ones. Henceforth, each of the positive tiles was paired with its corresponding binary vector to constitute a training pair that represents a specific phantom. Then, to accurately predict the hot nodes, each of the positive tiles was associated with a multi-linear regression model *R_i_*. All linear regression models *R_i_* corresponding to all the positive tiles were trained using a least square minimization technique across all training pairs. Finally, the prediction of the hot nodes was obtained by an intersection over union (IOU) of the hot nodes predicted by all MLRs.

## 3. Results

### 3.1. Evaluation of the Functional A Priori Mask Recovery

**Figure 5:**
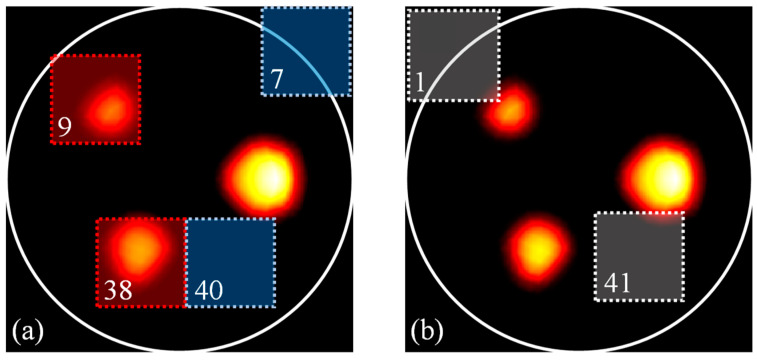
ML-based delineation of the region of interest. (**a**) Representative positive and negative tiles shown in red and blue, respectively. (**b**) Representative false negative tiles. The number in the bottom left corner indicates the index of the tile in a 7 *×* 7 tile^2^ division scheme.

The performance evaluation of our AI-based functional a priori mask recovery was first conducted using a simulation study. Here, a 7 × 7 tiles^2^ division of the temperature image was used. Considering the size 256 × 256 pixel^2^ of the temperature maps, the resulting size of the sub-images was 64 × 64 pixels^2^. The neural network was trained using 75% of the total dataset, while 25% of it was used for its validation. For the testing, 1073 new cases were created. The ML-based tile classification used to detect regions of interest with significant signal within the MRT image showed an accuracy of ~97%. Figure 5a shows the representative tiles that were correctly classified as positive in red and negative in blue. As can be seen, the positive tiles indeed captured the regions with higher temperature increase, as can be seen with tiles 9 and 38. It also showed negative tiles 7 and 40, which allowed us to discard the regions where no change in temperature occurred. However, a 3% error in accuracy was still observed. This error was uniquely due to positive tiles that were classified as negative (false negative). Figure 5b shows tiles 1 and 41, which are two representative false negative tiles. These tiles were classified as negative since only a small part of the inclusions was within them. Although it seems as if this part was only due to heat diffusion, it actually contained a single hot node and should have been classified as a positive tile. Nevertheless, no false positives were observed in the entire dataset.

**Figure 6:**
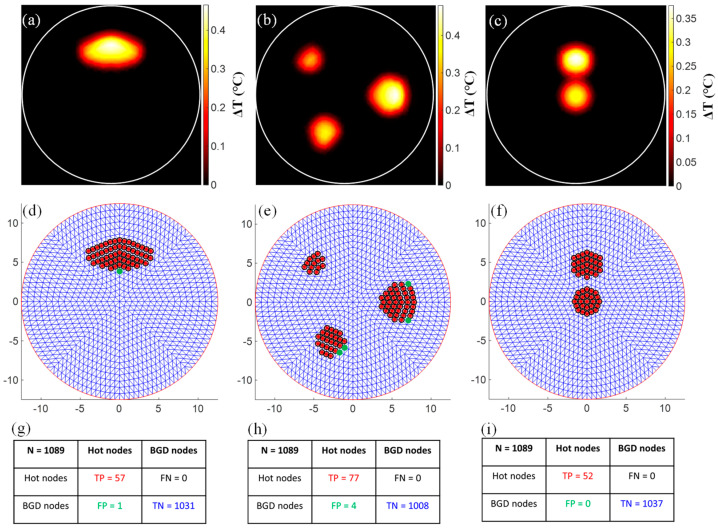
Results obtained on three representative cases using our new AI-based algorithm. (**a**–**c**) Temperature maps. (**d**–**f**) The predicted hot nodes are presented with red asterisks. The ground truth hot nodes are represented with black circles. The cold nodes incorrectly predicted as hot nodes are marked with green dots. (**g**–**i**) Confusion matrix: true positive (TP), true negative (TN), false positive (FP), and false negative (FN).

At the output of the ML-based tile classification algorithm, the positive tiles were identified and fed into the second step of our algorithm, where the detection of the hot nodes was made. On the other hand, the negative tiles were identified and discarded. This significantly improved the robustness of the hot node detection algorithm and drastically reduced its computation time. For each of the positive tiles, a dedicated MLR model was implemented and trained as described in Section 2.3.2. These MLR models were then tested on the entire set of 1073 testing images. The results obtained on three representative cases are presented in Figure 6. Case 1 contains an elliptical inclusion centered at (0,6) and has a semi-major and semi-minor axis equal to 4.5 mm and 2 mm, respectively. Case 2 is the same phantom presented in Figure 2 and contains three circular inclusions respectively centered at (−5,5), (−3,−5), and (6,0). The inclusions have a radius of 1.5 mm, 2 mm, and 2.5 mm, respectively. Case 3 contains two inclusions. The first one has a radius of 1.5 mm and is centered at the center of the phantom. The second inclusion is 2 mm in diameter and was intentionally placed only 1.5 mm (edge-to-edge) away from the first one at (0,5) to test for the closest separation that could be resolved by our algorithm. The temperature maps measured at the three phantoms are presented in the first row, Figure 6a–c. The second row depicts the results obtained using our hot node detection algorithm. Here, the red asterisks show the predicted hot nodes, while the corresponding ground truth hot nodes are represented by black circles. The cold nodes that were mistakenly predicted as hot nodes are marked with green dots. The third row shows the confusion matrix corresponding to each of the cases. Firstly, we calculated the number of nodes that were correctly predicted as hot nodes or not. These are respectively referred to as the true positive (TP) and true negative (TN) nodes. Then, we calculated the false positive (FP) and false negative (FN), which are respectively the nodes incorrectly predicted as hot nodes and the true hot nodes missed by our algorithm. Our algorithm performed highly in these three cases and predicted 100% of all true hot nodes. Again, it could be observed that the number of false negative nodes was null (FN = 0) in all three cases (Figure 6g–i). This resulted in a sensitivity of 100%. In other words, all the hot nodes were successfully detected. This is very important since it means that the entire tumor was correctly delineated. Note that the FN nodes represent the most undesirable error since they correspond to parts of the lesion that will be misdetected and thus not treated or surgically removed.

**Figure 7:**
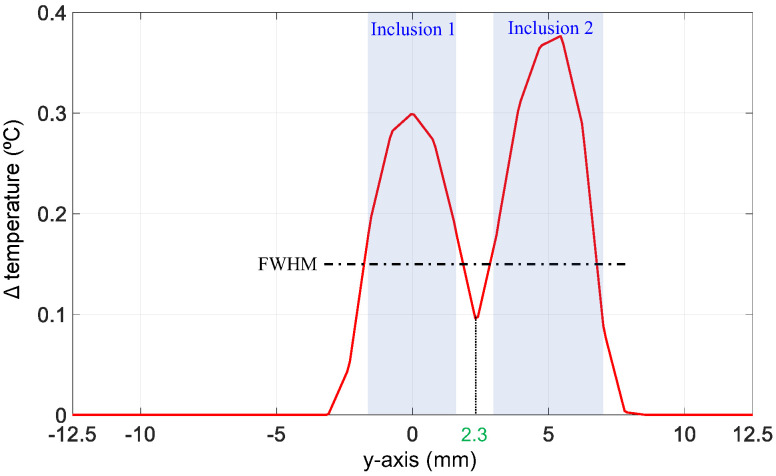
Temperature profile along the y-axis passing through the center of the two inclusions of Case 3. FWHM, full width at half maximum.

On the other hand, our algorithm slightly overestimated the number of hot nodes in Cases 1 and 2, as can been seen in Figure 6d,e. These nodes were incorrectly predicted as hot nodes and represent the false positive (FP) nodes in these cases, as summarized in their corresponding confusion matrix (Figure 6g,h). Nevertheless, one can observe that these FP nodes were the closest mesh neighbors to the real hot nodes. These were at an average distance less than 0.6 mm from the real hot nodes, and thus represent a neglectable error compared to the poor resolution of diffuse optical imaging. In Case 3, the algorithm performed perfectly and did not show any errors, although the inclusions were intentionally positioned close to each other. Even the single node separating the two inclusions was not mistakenly predicted as a hot node. This is in accordance with our previous findings using resolution phantoms with several side-by-side inclusions [40]. Technically, inclusions can be correctly separated as long as the temperature between them is below the full width at half maximum (FWHM) of the temperature at the inclusion with the lowest temperature increase. By plotting the profile along the y-axis, we observed that the temperature change at this node located at (0,2.3) between the two inclusions was below the FWHM of the temperature at inclusion 1, as shown in Figure 7.

### 3.2. PMI Reconstruction with vs. without A Priori Information

**Figure 8:**
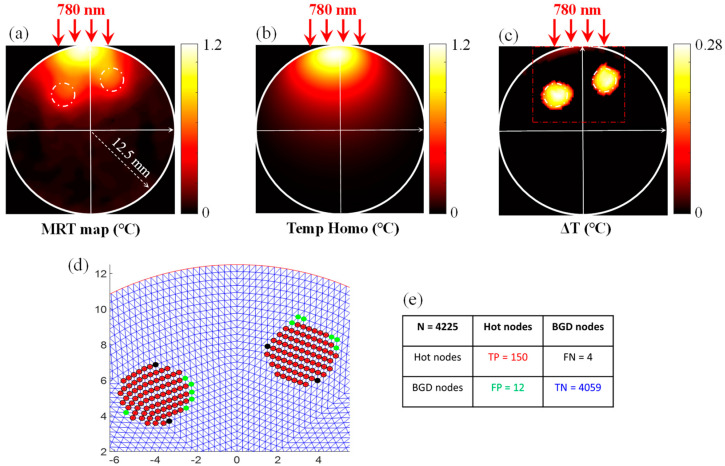
(**a**) The MRT temperature map measured after heating the phantom for 12 s. The inclusions are delignated with a white dot-dashed-line. The laser illumination is depicted with red arrows. (**b**) Temperature map obtained on the fitted homogenous phantom. (**c**) The difference in temperature between the MRT and the homogenous phantom. (**d**) Results of the hot node prediction process. The results are presented in the dot-dash box shown in panel (**e**). The predicted hot nodes are presented with red asterisks. The ground truth hot nodes are represented with black circles. The cold nodes incorrectly predicted as hot nodes are marked with green dots. The hot nodes incorrectly predicted as cold nodes are marked with black dots.

After successfully evaluating the performance of our algorithm on numerical phantoms, its performance was tested on experimental MRT data. For this experimental study, the absorption coefficient of the agarose phantom was adjusted using a black ink dye and was set to 0.01 mm^−1^. Two 4-mm diameter inclusions were embedded into the agarose phantom (Figure 8a).

Their absorption coefficient was increased to 0.023 mm^−1^ to mimic the higher absorption of cancerous tissue [41]. The first inclusion was located at (−4,5.25) and the second one at (3.1,7.5), which was slightly lower than the first one. Here, the heating was performed by only illuminating the phantom from its upper surface. The laser-induced temperature was measured using MRT. The MRT temperature map measured after heating the phantom for 12 s is presented in Figure 8b.

As previously mentioned, the phantom was only illuminated from its top surface. Consequently, the MRT temperature map showed a high increase in temperature under the illumination site. During the preprocessing, fitting near the laser area was performed to recover the average homogenous optical absorption of the phantom, which was used to generate the homogenous temperature map. Then, the latter was subtracted from the MRT temperature map to eliminate the high increase in temperature under the laser spot, as previously shown (Figure 8c). The resulting difference in temperature increase was then fed into our hot node prediction algorithm.

The results obtained using our algorithm on this phantom are presented in Figure 8d. In this figure, the red asterisks show the hot nodes that were correctly predicted by our method. The corresponding ground truth hot nodes are represented by black circles. As can be seen, our method predicted most of the hot nodes with a sensitivity over 97%. The sensitivity was degraded by the misclassification of four hot nodes (FN = 4), as shown with the black dot in Figure 8d and the confusion matrix presented in Figure 8e. Nevertheless, these nodes represent the closest neighbors. Since a fine mesh was also used, these were located at a distance less than 0.15 mm. The effect of this error will not affect the reconstruction quality since it will not be employed as a hard, but as soft a priori [42]. In this case, our algorithm also slightly overestimated the number of hot nodes, as shown with green dots in Figure 8d,e. These nodes were wrongly predicted as hot nodes and represent the false positive (FP) nodes in these cases, as summarized in their corresponding confusion matrix (Figure 8e). Similarly, these FP nodes were located at an average distance less than 0.15 mm from the real hot nodes and represent their closest mesh neighbors.

**Figure 9:**
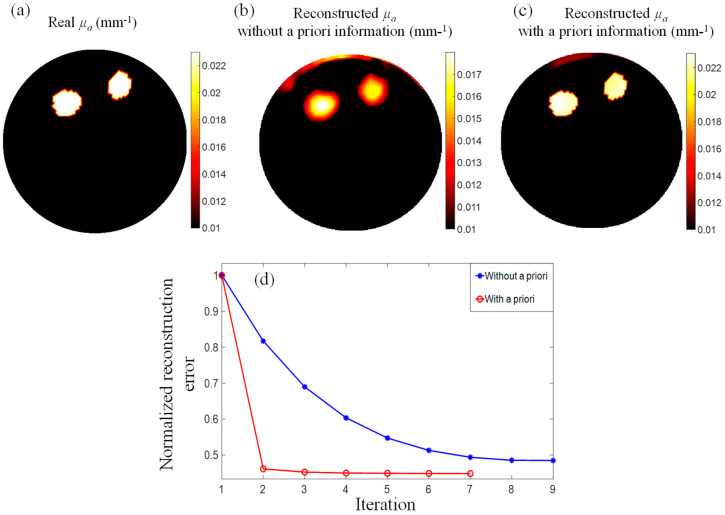
(**a**) The real absorption map. The PMI reconstructed absorption maps obtained (**b**) without, and (**c**) with the use of the penalty matrix. (**d**) Image reconstruction convergence errors obtained when reconstructing the image with (red), and without (blue) the use of the penalty matrix.

The MRT temperature map was then fed into the PMI image reconstruction algorithm to reconstruct the absorption map of the phantom (Figure 9). To evaluate the use of the a priori information calculated using our new algorithm, a comparison between the reconstruction results obtained without and with a priori information was performed. Firstly, absorption maps of the phantom were obtained using the standard PMI image reconstruction algorithm without a priori using Equation (4) (Figure 9b). Secondly, the predicted hot nodes were used to generate the penalty matrix described in Equation (6). This penalty matrix was then implemented in Equation (5) to reconstruct absorption maps using the information retrieved using our new algorithm (Figure 9c).

The absorption maps reconstructed with and without a priori information showed that both inclusions had been accurately localized, independently of their depth or location within the phantom. However, the use of the a priori information showed a clear improvement in the spatial resolution of the obtained absorption map. In addition, the higher performance of our new algorithm was shown by the quantification accuracy of the recovered absorption coefficient values. Using the standard PMI image reconstruction algorithm, the average error of the recovered absorption coefficient was as low as 31.7% for inclusion 1 and 35.6% for inclusion 2. When using the a priori information, the average error was decreased to 2.3% and 4.1% for inclusion 1 and inclusion 2, respectively. This improvement in the quality of the recovered absorption coefficient is directly attributed to constraining the PMI inverse problem using the penalty matrix obtained with our new method. The improvement due to the use of the a priori information could also be observed in the background region outside the inclusions. Indeed, one could observe some reconstruction artifacts when the standard reconstruction was performed (Figure 9b). Although they were close to the surface and only represented less than 26.5% of the inclusions’ maximum absorption, these artifacts were further reduced to 11.7% when using the a priori information. Table 1 summarizes the mean and standard deviation of the recovered absorption coefficient values.

In addition, the iterative reconstruction process was stopped if the reconstruction error calculated at a given iteration was greater than the previous one. To avoid local minima, the algorithm was allowed to recalculate an update using Equations (4) or (5) for three times before stopping it. As can be seen in Figure 9d, the algorithm ran for nine and seven iterations when not using and then using the a priori information, respectively. More importantly, we could observe that the algorithm rapidly converged at the first iteration when using the a priori information. Then, it continued to converge slowly to a minimum of 44%. On the other hand, the standard algorithm showed a slower convergence rate and reached a minimum of 49% after only nine iterations. Considering that the time necessary for each iteration is around 11 min, using the a priori information allowed us to accelerate the image reconstruction process approximately 9-fold.

## 4. Conclusions

In the last few years, PMI has been tested extensively on tissue-like phantoms and ex vivo samples bearing different tumor mimicking heterogeneities. Our standard PMI image reconstruction algorithm showed high performance in recovering spatially-resolved absorption maps with high resolution and quantitative accuracy compared to other conventional diffuse optics imaging modalities such as DOT.

The standard PMI image reconstruction process is first initialized by generating a homogenous temperature map using the PMI forward solver by considering a homogenous distribution of the optical absorption of the medium. Then, this homogenous temperature is compared to the measured MRT temperature map, and the difference between these two temperature maps is minimized iteratively by updating the assumed optical absorption. At each iteration, Jacobian matrices are calculated for each FEM node via the perturbation theory. While this approach provides accurate results, its computational process is very time consuming. Thus, improving the convergence of the minimization process reduces the number of reconstruction iterations, which in turn reduces the overall image reconstruction time.

In this paper, we proposed a new AI-based image reconstruction algorithm and evaluated its performance in generating fast and accurate absorption maps of a phantom bearing two tumor-like inclusions. In this approach, the PMI standard algorithm was improved by incorporating a supervised machine learning approach. This approach detects the tumor margins directly from the MRT temperature maps. The detected tumor margins are then used to implement a binary mask used to build a penalty matrix for the preconditioning of the minimization process. In fact, the implemented penalty matrix is used in the standard PMI algorithm as soft functional a priori information to enhance and accelerate the resolution of the PMI inverse problem and the absorption map recovery process. The obtained results show the successful use of this approach by not only accelerating the image reconstruction process by approximately 9-fold, but also reducing the average error of the recovered absorption coefficient by around 30% compared to the standard PMI method. Finally, we demonstrated that combining novel AI-based methodologies and the PMI image reconstruction algorithm has the potential to provide faster detection and diagnosis, thus paving the way for preclinical and clinical research.

## Figures and Tables

**Table 1:** Mean and standard deviation of the recovered absorption coefficient values (mm^−^^1^) of inclusion 1 and inclusion 2.

	Real	Reconstructed without A Priori Information	Reconstructed with A Priori Information
Inclusion 1	0.023	0.0157 ± 0.00140	0.0226 ± 0.00030
Inclusion 2	0.023	0.0148 ± 0.00130	0.0220 ± 0.00026
Background	0.010	0.0094 ± 0.00089	0.0100 ± 0.00040

## Data Availability

The data used in this study are available upon request from the corresponding author.
